# Hesitancy and reactogenicity to mRNA-based COVID-19 vaccines–Early experience with vaccine rollout in a multi-site healthcare system

**DOI:** 10.1371/journal.pone.0272691

**Published:** 2022-08-05

**Authors:** Sarah Al-Obaydi, Eileen Hennrikus, Nazar Mohammad, Erik B. Lehman, Abhishek Thakur, Taha Al-Shaikhly

**Affiliations:** 1 Department of Medicine, Division of Hospital Medicine, Penn State College of Medicine, Hershey, Pennsylvania, United States of America; 2 Department of Public Health Science, Penn State College of Medicine, Hershey, Pennsylvania, United States of America; 3 Harrisburg University of Science & Technology, Harrisburg, Pennsylvania, United States of America; 4 Department of Medicine, Division of Pulmonary, Allergy and Critical Care Medicine, Penn State College of Medicine, Hershey, Pennsylvania, United States of America; Universitas Syiah Kuala, INDONESIA

## Abstract

**Background:**

Hesitancy and incomplete vaccination against coronavirus disease 2019 (COVID-19) remains an obstacle to achieving herd immunity. Because of fear of vaccine reactions, patients with medical and allergic co-morbidities express heightened hesitancy. Limited information is available to guide these patients. We sought to identify factors associated with mRNA-based COVID-19 vaccines hesitancy and reactogenicity.

**Methods:**

We surveyed employees of a multi-site health system in central Pennsylvania who were offered the COVID-19 vaccine (N = 18,740) inquiring about their experience with the Moderna and Pfizer-BioNTech mRNA-based vaccines. The survey was administered online using the REDCap platform. We used multivariable regression analysis to determine whether a particular factor(s) (e.g., demographics, selected co-morbid allergic and medical conditions, vaccine brand, and prior COVID-19) were associated with vaccine reactogenicity including the occurrence and severity of local and systemic reactions. We also explored factors and reasons associated with vaccine hesitancy.

**Results:**

Of the 5709 who completed the survey (response rate, 30.4%), 369 (6.5%) did not receive the vaccine. Black race and allergy to other vaccines were associated with vaccine hesitancy. Reaction intensity following the first vaccine dose and allergic co-morbidities were associated with incomplete vaccination. Older individuals (>60 years) experienced less reactogenicity. Females had higher odds of local and systemic reactions and reported more severe reactions. Asians reported more severe reactions. As compared to Pfizer-BioNTech, the Moderna vaccine was associated with higher odds of vaccine reactions of higher severity. Prior COVID-19 resulted in more severe reactions following the first dose, but less severe reactions following the second dose.

**Conclusions:**

Targeted campaigns to enhance vaccination acceptance should focus on Black individuals, females, and those with allergic co-morbidities. Prior COVID-19 caused more severe reactions after the first but not the second vaccine dose. Moderna vaccine caused more vaccine reactions. Lessons learned from the early rollout of COVID-19 vaccine may serve to inform future novel vaccine experiences.

## Introduction

Soon after coronavirus disease 2019 (COVID-19) was declared a pandemic by the World Health Organization in early 2020, the illness severity and its negative psychological and economic impact on individuals and societies worldwide made it clear that herd immunity through vaccination is the ultimate approach to curb down the spread and bring life back to normal [1–3]. As scientists across the globe raced to develop an effective vaccine, mRNA-based vaccine technology offered an unprecedented platform for rapid and successful development of two anti-COVID-19 vaccines namely Pfizer-BioNTech (BNT162b2) and Moderna (Mrna-1273) vaccines [4–6]. Based on clinical trial results demonstrating remarkable efficacy for these two vaccines in preventing COVID-19 illness [4, 5], the United States Food and Drug Administration issued emergency use authorization for these two vaccines in late 2020.

Although the Pfizer-BioNTech and Moderna vaccines demonstrated good safety profiles in clinical trials [4, 5], the rapid pace of their development along with media and internet outlets highlighting serious adverse reactions, vaccine hesitancy became a growing concern [7, 8]. In a survey of 991 US adults, 10.8% were not intending to receive the COVID-19 vaccine citing the need for more information about the vaccine, and the lack of trust [9]. Another survey study of healthcare workers from two academic centers in Philadelphia, Pennsylvania, explored factors associated with vaccine hesitancy among healthcare providers before vaccine rollout and concluded that Black race and female sex were associated with higher odds of vaccine hesitancy [10]. According to the US Centers for Disease Control and Prevention (CDC), as of February 8^th^, 2022, 75.7% of the eligible US population is vaccinated [11].

As fear of side effects can magnify vaccine hesitancy, identifying factors that may influence the severity of vaccine reactions is of particular importance. Clinical trial data suggested that younger age groups experience more severe reactions [4]. The CDC vaccine safety report and systemic review of clinical trials implied Moderna vaccine causes more reactogenicity [12]. However, the impact of other host factors such as race, ethnicity, and co-morbid allergic and medical conditions on vaccine reactogenicity is not well examined. Knowledge of such interactions may assist in overcoming barriers to vaccination.

In this online survey, we took advantage of a large population of vaccine-eligible adults employed by a multi-site healthcare system in central Pennsylvania and explored factors associated with vaccine hesitancy and reactogenicity following the first and second doses of the Moderna and Pfizer-BioNTech vaccines, in the first phase of vaccine roll-out in early 2021.

## Materials and methods

### Study design

We conducted a survey-based cross-sectional study of Penn State Health employees to explore barriers to vaccination and their experience with the Moderna and Pfizer-BioNTech vaccines. Penn State health encompasses four major medical centers across central Pennsylvania (Hershey Medical Center, St. Joseph Hospital, the Medical Group, and Holy Spirit Hospital). The study was approved by the Institutional review board at the Penn State College of Medicine. Anonymous electronic survey designed using REDCap, an electronic data capture tool, was distributed via email on February 23, 2021, with a follow-up reminder on March 1, 2021. At the time of survey distribution, all employees were being offered either the Pfizer-BioNTech or Moderna vaccine depending upon availability and were considered eligible to participate.

### Data captured by the survey

After e-consenting to participation, we collected information on participants’ demographics (age, sex, race, ethnicity, occupation), co-morbid allergic (allergic rhinitis, asthma, food allergy, drug allergy, stinging insect allergy, and having an epinephrine auto-injector prescription), and medical conditions (heart diseases, pulmonary diseases other than asthma, rheumatological diseases, diabetes, and neurological diseases), prior COVID-19 (defined as having positive testing for COVID-19 before vaccine administration whether individuals were symptomatic or asymptomatic), and the occurrence and severity of local or systemic reactions. Local reactions were defined as pain, swelling, or redness at the vaccine injection site. All other reactions were considered systemic reactions. The severity of the reaction was captured on a 1–10 scale and no guidance was offered to the participants regarding using the severity scale. For participants who elected not to receive the vaccine, follow-up questions were presented to them to explore reasons for opting out of receiving the vaccine. A list of survey questions is provided in S2 File.

### Study groups

Participants were classified into two groups (vaccine receivers and non-receivers) based on whether they received the first dose of either the Pfizer-BioNTech or Moderna vaccine. Vaccine receivers were subdivided into the Pfizer-BioNTech and Moderna subgroups and were also categorized by the presence or absence of 1) prior COVID-19, and 2) different co-morbid allergic and medical diagnoses.

### Outcomes

Among vaccine non-receivers, we explored reason(s) for declining vaccination. Among vaccine receivers, we determined factors associated with the occurrence and severity of local and systemic reactions after the first and second vaccine doses. We explored age, sex, race, ethnicity, vaccine brand, co-morbidities, and reactions to the first or second vaccine doses as potential predictors.

### Statistical analysis

The number (N) and percentage (%) were used to describe categorical variables. The median and interquartile range (IQR) were used to describe continuous ranked variables. Chi-square tests assessed differences in the occurrence of local and systemic reactions among the different groups. A Mann-Whitney *U* test or a Kruskal Wallis test assessed differences in severity of local or systemic reaction severity among the different groups. Multivariable binomial logistic regression analysis assessed factors associated with the occurrence of vaccine reactions. Model-adjusted odds ratios (OR) quantified the magnitude and direction of significant associations. The Hosmer-Lemeshow test assessed the goodness of fit for logistic regression models. Multivariable quantile regression of the median was used to examine factors associated with the severity of vaccine reactions. Model-adjusted estimates of the differences of group medians quantified the magnitude and direction of significant differences. Predictors were checked for multicollinearity prior to multivariable modeling using variance inflation factors statistics, but no multicollinearity was found. For analysis purposes, individuals identified as American Indian, Alaska Native, Native Hawaiian or Other Pacific Islander, and those who did not disclose their race or elected “other race” and multi-racial individuals were grouped as other race. A priori alpha criterion of 0.05 was used to establish statistical significance. Analyses were performed using SAS version 9.4 (SAS Institute, Cary, NC).

## Results

### Vaccine receivers versus non-receivers

**Fig 1:**
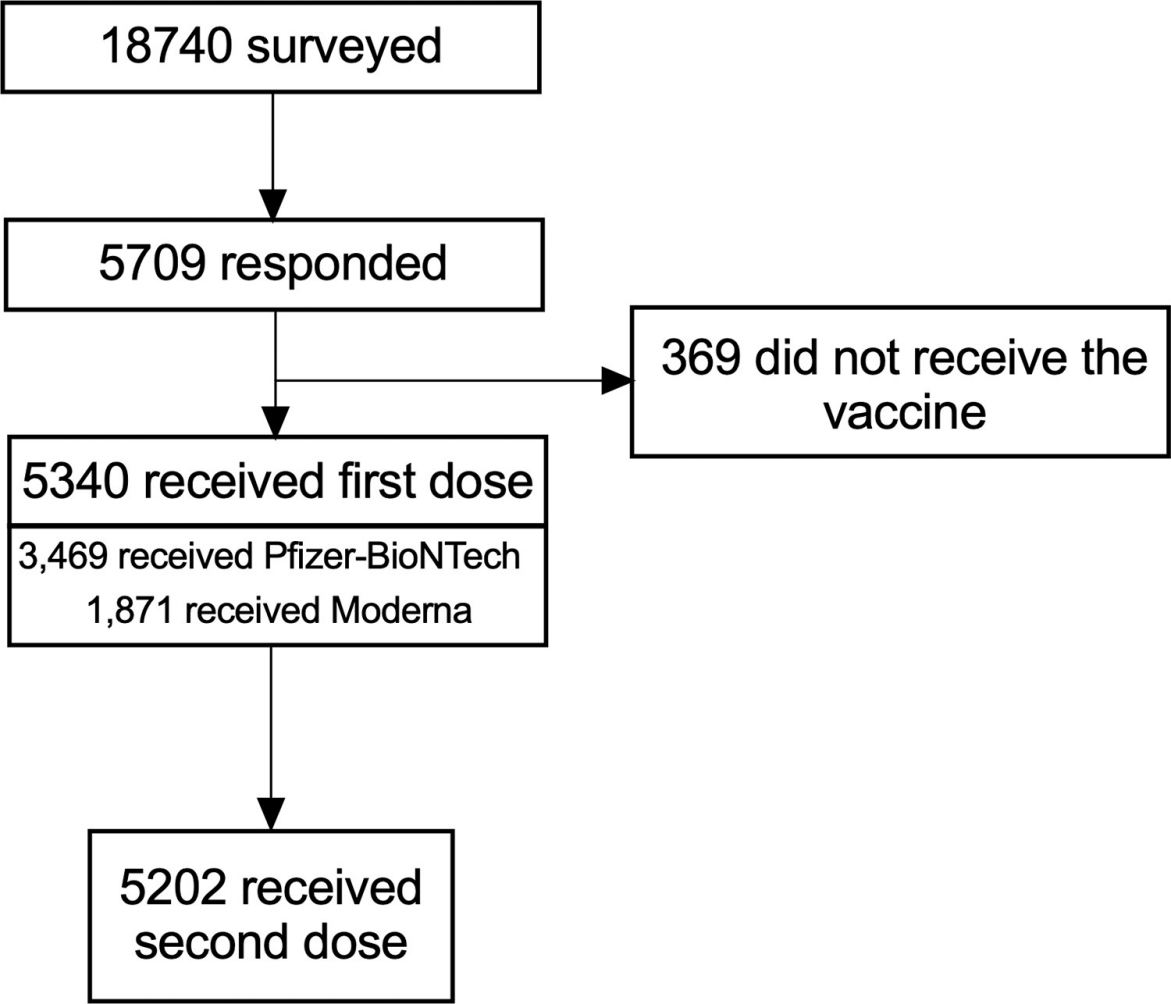
Study participants. Shown is a flowchart summarizing the breakdown of study participants.

Out of 18,740 employees surveyed, 5,709 completed the survey (response rate, 30.4%) (**Fig 1**). Baseline characteristics of vaccine receivers (N = 5340) and non-receivers (N = 369) are contrasted in **S1 Table**. In logistic regression analysis, younger age group (18–24 years), Black race, female sex, non-clinical occupation, allergy to other vaccines, and asthma were independent factors associated with higher odds for opting out of vaccination, whereas Asian individuals were more likely to opt-in for vaccination (**Table 1**). The most common reasons cited for electing not to receive the vaccine were concerns about side effects (57.5%) and doubts about vaccine effectiveness (20.3%) (**S2 Table)**. Among vaccine receivers, 3,469 received the Pfizer-BioNTech and 1,871 received the Moderna, based on vaccine availability. Age and sex distributions were different but allergic and medical co-morbidities were similar (**S3 Table**).

**Table 1:** Factors associated with vaccine hesitancy.

Characteristics	OR (95% CI)	*p* value
Age (yrs.), 25–39 vs. 18–24	1.05 (0.64, 1.81)	0.864
Age (yrs.), 40–59 vs. 18–24	0.87 (0.53, 1.51)	0.595
Age (yrs.), 60 plus vs. 18–24	0.48 (0.26, 0.91)	**0.022**
Sex, female vs. male	1.63 (1.19, 2.27)	**0.003**
Race, Black vs. White	2.86 (1.59, 4.87)	**<0.001**
Race, Asian vs. White	0.39 (0.16, 0.79)	**0.018**
Race, Other vs. White	1.18 (0.58, 2.21)	0.624
Hispanic ethnicity	1.26 (0.64, 2.31)	0.479
Occupation^a^, non-clinical vs. clinical	1.61 (1.23, 2.12)	**0.001**
Occupation^a^, unable to classify vs. clinical cclinicalclinical	1.34 (0.98, 1.82)	0.062
Food allergy	1.39 (0.95, 1.97)	0.079
Drug allergy	0.91 (0.69, 1.19)	0.515
Bee sting allergy	0.99 (0.56, 1.64)	0.967
Allergy to other vaccines	7.46 (4.38, 12.47)	**<0.001**
Epinephrine prescription	1.24 (0.71, 2.08)	0.429
Heart diseases	1.18 (0.44, 2.62)	0.708
Asthma	1.41 (1.02, 1.90)	**0.032**
Other lung diseases (e.g., COPD)	0.31 (0.02, 1.61)	0.271
Rheumatological diseases	1.13 (0.71, 1.72)	0.599
Neurological diseases	1.84 (0.82, 3.64)	0.105
Diabetes	1.06 (0.63, 1.71)	0.812

The adjusted odds ratio (OR) and 95% confidence interval (CI) are presented. For categorical variables with more than two categories, the reference category is indicated.

^a^The clinical category included physicians, residents and fellow physicians, advanced practice providers, and nurses; the non-clinical category included researchers, technicians, support staff, administration, maintenance, security, care coordination, social workers, and IT staff; the unable to classify category included students, others, and unknown.

### Local and systemic reactions after first vaccine dose

**Fig 2:**
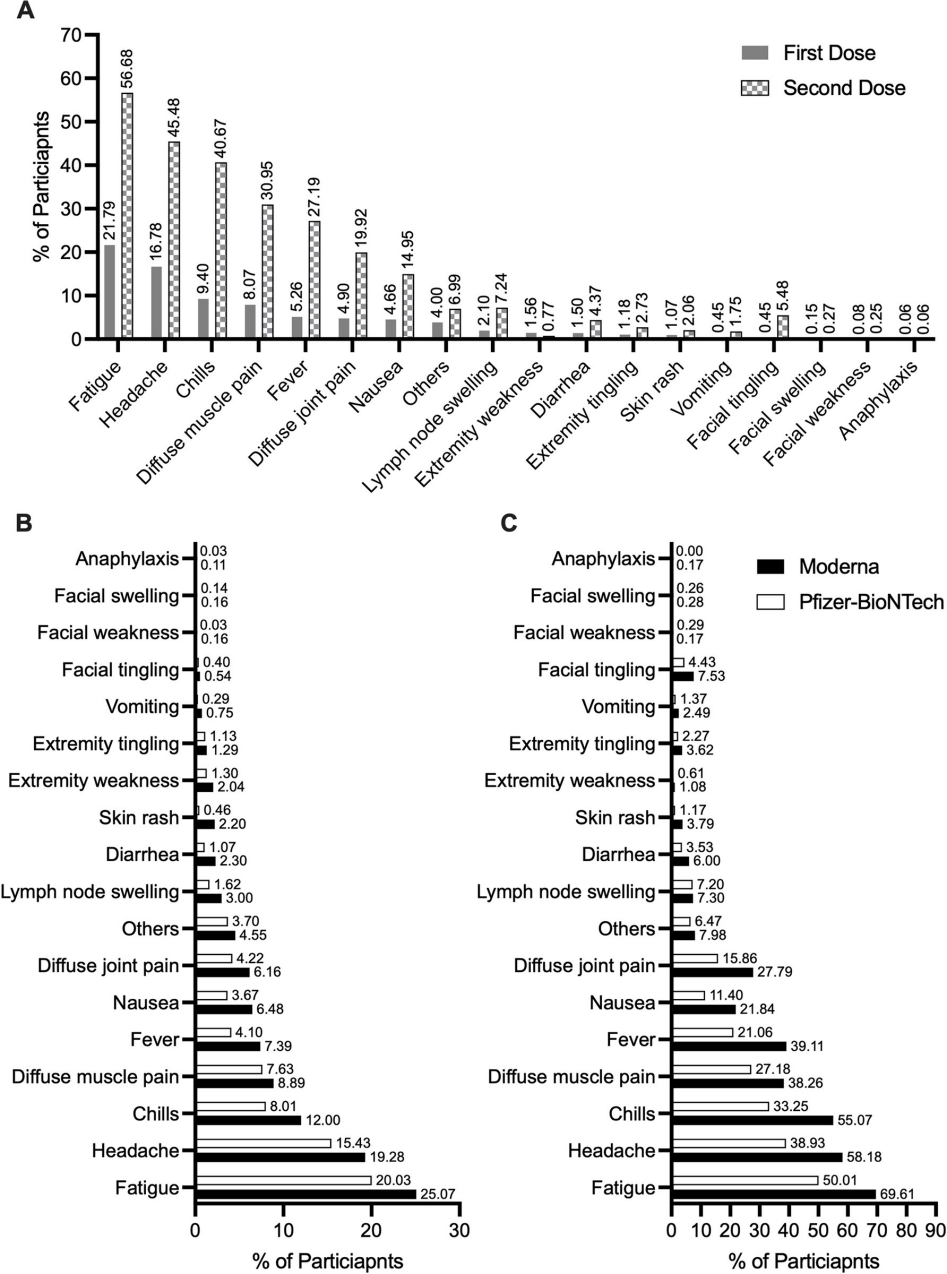
Frequency of systemic reactions after the first and second vaccine dose. (A) Frequency of systemic reactions after the first and second vaccine dose across the full study population (B) Frequency of systemic reactions following the first vaccine dose by vaccine brand. (C) Frequency of systemic reactions following the second vaccine dose by vaccine brand.

Of the 5,340 participants who received the first vaccine dose, 5,330 responded to whether they developed a local reaction and 79.9% (N = 4261/5,330) indicated that they had a local reaction with a median severity of four (IQR, 3 to 6, N = 4,258; missing, 3). Only 31.3% (N = 1665/5,327; missing, 13) developed systemic reaction(s) with fatigue, headache, and chills being the most frequently reported (**Fig 2A**). Only 3 participants reported anaphylaxis. Systemic reactions were more common among Moderna versus Pfizer-BioNTech vaccine recipients (**Fig 2B**). The median severity of systemic reactions was five (IQR, 3 to 6, N = 1,656). Local reaction severity correlated with systemic reaction severity (p < 0.001) (**S1 Fig**). Logistic regression analysis showed that young age group (18–24 years), female sex, Moderna brand, and prior COVID-19 were independently associated with higher odds of local reactions after the first dose, whereas bee sting allergy and diabetes were associated with lower odds (**Table 2**). Factors associated with higher odds of developing systemic reaction after the first dose included female sex, Hispanic ethnicity, food allergy, allergy to other vaccines, having an epinephrine auto-injector, asthma history, Moderna brand, prior COVID-19, and first-dose local reactions (**Table 2**). Compared to younger participants (18–24 years), older individuals (>60 years) had lower odds of experiencing systemic reactions (**Table 2).** Focused analysis on allergic-type reactions (facial swelling, skin rash, or anaphylaxis) following the first dose showed that histories of allergy to other vaccines, asthma, and having an epinephrine auto-injector were significantly associated with allergic-type reactions (**Table 3**). Characteristics of the three study participants who developed anaphylaxis after the first vaccine dose are summarized in **S4 Table**.

**Table 2:** Factors associated with local and systemic reactions after the first COVID-19 vaccine dose.

	Local reaction		Systemic reaction	
Characteristic	OR (95% CI)	*p* value	OR (95% CI)	*p* value
Age (yrs.), 25–39 vs. 18–24	0.90 (0.61, 1.33)	0.604	1.05 (0.79, 1.39)	0.747
Age (yrs.), 40–59 vs. 18–24	0.66 (0.45, 0.98)	**0.038**	0.97 (0.73, 1.28)	0.808
Age (yrs.), 60 plus vs. 18–24	0.39 (0.26, 0.59)	**<0.001**	0.71 (0.51, 0.97)	**0.033**
Sex, female vs. male	1.31 (1.11, 1.55)	**0.001**	1.30 (1.11, 1.52)	**0.001**
Race, Black vs. White	1.14 (0.64, 2.04)	0.654	0.85 (0.51, 1.40)	0.515
Race, Asian vs. White	0.96 (0.70, 1.32)	0.818	1.12 (0.86, 1.46)	0.402
Race, Other vs. White	1.39 (0.82, 2.35)	0.221	1.17 (0.80, 1.73)	0.422
Hispanic ethnicity	1.02 (0.62, 1.68)	0.926	1.51 (1.02, 2.22)	**0.039**
Food allergy	1.17 (0.89, 1.55)	0.266	1.26 (1.00, 1.58)	**0.047**
Drug allergy	1.13 (0.95, 1.34)	0.183	1.08 (0.93, 1.26)	0.291
Bee sting allergy	0.66 (0.47, 0.93)	**0.017**	0.84 (0.61, 1.15)	0.274
Allergy to other vaccines	1.30 (0.57, 2.95)	0.536	2.12 (1.17, 3.84)	**0.014**
Epinephrine autoinjector	1.15 (0.76, 1.75)	0.514	1.56 (1.11, 2.20)	**0.011**
Heart diseases	1.27 (0.76, 2.12)	0.355	1.38 (0.88, 2.18)	0.163
Asthma	0.93 (0.74, 1.17)	0.525	1.39 (1.15, 1.68)	**0.001**
Other lung diseases	0.89 (0.41, 1.94)	0.775	1.03 (0.50, 2.15)	0.932
Rheumatological diseases	1.03 (0.77, 1.38)	0.854	1.11 (0.85, 1.44)	0.435
Neurological diseases	0.77 (0.44, 1.36)	0.37	0.89 (0.52, 1.52)	0.663
Diabetes	0.72 (0.54, 0.96)	**0.023**	0.88 (0.66, 1.16)	0.359
Moderna vs. Pfizer-BioNTech	1.95 (1.66, 2.29)	**<0.001**	1.26 (1.10, 1.43)	**0.001**
Prior COVID-19	1.60 (1.11, 2.30)	**0.011**	4.24 (3.28, 5.50)	**<0.001**
Local reaction post dose 1	-	-	1.96 (1.65, 2.34)	**<0.001**

The adjusted odds ratio (OR) and 95% confidence interval (CI) are presented. For categorical variables with more than two categories, the reference category is indicated. The Analysis is by binominal logistic regression.

**Table 3:** Allergic systemic reaction (ASR) and allergic co-morbidities.

	First Dose			Second Dose		
Characteristic	ASR (+)	ASR (-)	*p* value	ASR (+)	ASR (-)	*p* value
Food allergy	9 (13.4)	446 (8.5)	0.147	15 (12.5)	427 (8.4)	0.112
Drug allergy	18 (26.9)	1257 (23.8)	0.564	38 (31.7)	1206 (23.7)	**0.044**
Bee sting allergy	4 (6)	226 (4.3)	0.500	7 (5.8)	214 (4.2)	0.384
Allergy to other vaccines	5 (7.5)	45 (0.9)	**<0.001**	3 (2.5)	43 (0.8)	0.056
Epinephrine autoinjector	7 (10.4)	188 (3.6)	**0.003**	7 (5.8)	182 (3.6)	0.193
Asthma	13 (19.4)	595 (11.3)	**0.038**	18 (15)	565 (11.1)	0.183

Values are expressed as N (%). Statistical significance was assessed by the chi-square test. Abbreviation: ASR, allergic-type systemic reactions (skin rash, facial swelling, or anaphylaxis).

Using quantile regression of the median analysis, younger age groups (<60 years), female sex, Black race, Asian race, food allergy, Moderna brand, and prior COVID-19 were independent factors associated with more severe local reaction whereas Asians, epinephrine autoinjector carriers, and those with heart diseases, neurological diseases, or prior COVID-19 reported more severe systemic reactions (**Table 4**). Local and systemic reaction severity following the first dose by different participants’ attributes is summarized in **S5 Table**.

**Table 4:** Factors associated with the severity of local and systemic reactions following the first vaccine dose.

	Local reaction		Systemic reaction	
Characteristic	Mdn. dif. (95% CI)	*p* value	Mdn. dif. (95% CI)	*p* value
Age (yrs.), 18–24 vs. 60 plus	1.2 (0.76, 1.64)	**<0.001**	0 (-0.87, 0.87)	1.000
Age (yrs.), 25–39 vs. 60 plus	1.0 (0.81, 1.19)	**<0.001**	0 (-0.56, 0.56)	1.000
Age (yrs.), 40–59 vs. 60 plus	0.6 (0.38, 0.82)	**<0.001**	0 (-0.47, 0.47)	1.000
Sex, female vs. male	0.6 (0.40, 0.80)	**<0.001**	0 (-0.37, 0.37)	1.000
Race, Black vs. White	0.6 (0.05, 1.15)	**0.034**	1 (-0.39, 2.39)	0.158
Race, Asian vs. White	0.6 (0.23, 0.97)	**0.001**	1 (0.08, 1.92)	**0.033**
Race, Other vs. White	0 (-0.55, 0.55)	1.000	1 (-0.07, 2.07)	0.067
Hispanic ethnicity	0 (-0.83, 0.83)	1.000	0 (-0.88, 0.88)	1.000
Food allergy	0.4 (0.06, 0.74)	**0.020**	0 (-0.44, 0.44)	1.000
Drug allergy	0 (-0.18, 0.18)	1.000	0 (-0.43, 0.43)	1.000
Bee sting allergy	0 (-0.43, 0.43)	1.000	0 (-0.83, 0.83)	1.000
Allergy to other vaccines	-0.2 (-1.6, 1.2)	0.779	0 (-0.92, 0.92)	1.000
Epinephrine autoinjector	-0.4 (-0.86, 0.06)	0.085	1 (0.29, 1.71)	**0.006**
Heart diseases	0.4 (-0.14, 0.94)	0.147	1 (0.18, 1.82)	**0.017**
Asthma	0 (-0.24, 0.24)	1.000	0 (-0.45, 0.45)	1.000
Other lung diseases (e.g., COPD)	-0.4 (-1.84, 1.04)	0.586	1 (-1.12, 3.12)	0.356
Rheumatological diseases	0.2 (-0.35, 0.75)	0.472	0 (-0.79, 0.79)	1.000
Neurological diseases	0 (-1.19, 1.19)	1.000	2 (0.32, 3.68)	**0.019**
Diabetes mellitus	0 (-0.43, 0.43)	1.000	0 (-0.65, 0.65)	1.000
Moderna vs. Pfizer-BioNTech	0.8 (0.57, 1.03)	**<0.001**	0 (-0.39, 0.39)	1.000
Prior COVID-19	1 (0.63, 1.37)	**<0.001**	1 (0.41, 1.59)	**0.001**
Local reaction post Dose 1	-	**-**	0 (-0.40, 0.40)	1.000

The adjusted Mdn. dif. (Median difference) and 95% confidence interval (CI) are presented. For baseline characteristics with more than two categories, the reference category is indicated. The analysis is by quantile regression of the median.

### Local and systemic reactions following the second vaccine dose

Of those who received the first dose (N = 5340), only 132 participants (2.5%) indicated that they did not complete their second dose. The two most cited reasons were: 1) the second dose not due yet (68.9%), and 2) the intensity of reaction following the first dose (9.8%) (**S2 Table**). After excluding those whose second dose was not due at the time of the survey (N = 91), Moderna brand, first-dose vaccine reactions (fatigue, allergic-type systemic reactions such as rash, facial swelling, or anaphylaxis), and post-Dose 1 COVID-19 were independent factors associated with incomplete vaccination (**Table 5**). Among the 3 participants who reported anaphylaxis to the first vaccine dose, only one opted to receive the second dose and reported having anaphylaxis after receiving the second dose. Incompletely vaccinated participants reported more intense systemic (p < 0.001), but not local (p = 0.099) reactions compared to fully vaccinated individuals.

**Table 5:** Factors associated with incomplete vaccination.

Characteristic	OR (95% CI)	*p* value
Age group	1.45 (0.93, 2.26)	0.101
Sex, female vs. male	2.15 (0.73, 6.39)	0.167
Moderna vs. Pfizer-BioNTech	4.00 (1.92, 8.36)	**<0.001**
Local reaction post Dose 1	0.65 (0.27, 1.56)	0.338
Fever	1.99 (0.54, 7.29)	0.299
Chills	0.69 (0.21, 2.35)	0.558
Headache	1.31 (0.54, 3.17)	0.548
Fatigue	4.08 (1.68, 9.90)	**0.002**
Nausea	0.14 (0.02, 0.97)	**0.046**
Vomiting	6.94 (0.47, 103.57)	0.160
Diarrhea	0.67 (0.09, 5.12)	0.700
Diffuse muscle pain	0.49 (0.14, 1.74)	0.268
Diffuse joint pain	0.63 (0.15, 2.56)	0.513
Lymph node swelling	3.76 (1.21, 11.72)	**0.022**
Extremity tingling/numbness	2.24 (0.39, 12.99)	0.369
Facial tingling/numbness	1.09 (0.20, 6.12)	0.919
Extremity weakness	2.21 (0.26, 18.93)	0.470
Facial weakness	-	-
Allergic reaction (facial swelling, rash, or anaphylaxis)	10 (3.85, 26)	**<0.001**
COVID-19 Post Dose 1	22.96 (7.08, 74.4)	**<0.001**

The adjusted odds ratio (OR) and 95% confidence interval (CI) are presented. The analysis is by binominal logistic regression. The age group was treated as an ordinal variable for purpose of this analysis.

Among second dose receivers (N = 5,202; missing, 6), 5,194 participants responded to whether they developed a local reaction and 78.1% (N = 4,064/5,194) indicated that they developed a local reaction with a median severity of four (IQR, 3 to 6, N = 4062; missing, 2) and 5,196 responded to whether they developed a systemic reaction and 70% (N = 3,644/5,196) indicated that they developed a systemic reaction with a median severity of six (IQR, 4 to 8; N = 3,633; missing, 11). Only three participants reported anaphylaxis following the second dose, one of whom also reported anaphylaxis to the first dose of the vaccine **(Fig 2A**). Systemic reactions were more common following the second dose and following the Moderna vaccine as compared to the first dose and Pfizer-BioNTech vaccine respectively (**Fig 2C**). There were significant correlations between the local and systemic reactions after the first and second vaccine dose (p < 0.001) (**S1 Fig**). Regarding factors associated with reaction occurrence following the second dose, and whether local or systemic reactions after the first or second dose influenced reactogenicity, the following factors were independently associated with higher odds of local reactions after the second dose: female sex, Asian race, Moderna brand, and first-dose local reaction. Prior COVID-19 was associated with lower odds of local reactions (**Table 6**). Higher odds of systemic reactions were noticed among individuals 25–39 years old, females, and those with food allergy, drug allergy, or those who experienced first-dose systemic reactions or second-dose local reactions (**Table 6**). Moderna brand was associated with higher odds of systemic reactions after the second vaccine dose. Older participants (>60 years), and Blacks had lower odds of developing second-dose systemic reactions (**Table 6**). Focused analysis on allergic-type reactions (facial swelling, skin rash, or anaphylaxis) showed that a history of drug allergy was significantly associated with second-dose allergic-type reactions (**Table 3**). Characteristics of the three study participants who developed anaphylaxis after the second vaccine dose are summarized in **S4 Table**.

**Table 6:** Factors associated with local and systemic reactions after second dose of the COVID-19 vaccine.

	Local reaction		Systemic reaction	
Characteristic	OR (95% CI)	*p* value	OR (95% CI)	*p* value
Age (yrs.), 25–39 vs. 18–24	0.92 (0.61, 1.38)	0.680	1.47 (1.07, 2.02)	**0.019**
Age (yrs.), 40–59 vs. 18–24	0.85 (0.57, 1.28)	0.438	1.06 (0.77, 1.45)	0.739
Age (yrs.), 60 plus vs. 18–24	0.83 (0.54, 1.29)	0.407	0.66 (0.47, 0.92)	**0.015**
Sex, female vs. male	1.21 (1.00, 1.45)	**0.049**	1.68 (1.44, 1.96)	**<0.001**
Race Black vs. White	0.77 (0.43, 1.39)	0.384	0.45 (0.27, 0.74)	**0.002**
Race Asian vs. White	1.59 (1.08, 2.33)	**0.018**	1.08 (0.80, 1.45)	0.626
Race Other vs. White	1.40 (0.80, 2.44)	0.244	0.67 (0.44, 1.03)	0.066
Hispanic ethnicity	0.74 (0.45, 1.24)	0.252	1.36 (0.86, 2.16)	0.189
Food allergy	1.01 (0.75, 1.35)	0.964	1.31 (1.00, 1.70)	**0.049**
Drug allergy	1.03 (0.85, 1.24)	0.795	1.30 (1.11, 1.54)	**0.002**
Bee sting allergy	1.14 (0.76, 1.71)	0.535	0.87 (0.62, 1.24)	0.445
Allergy to other vaccines	1.14 (0.47, 2.72)	0.777	0.67 (0.32, 1.39)	0.281
Epinephrine autoinjector	0.93 (0.59, 1.47)	0.759	1.28 (0.84, 1.95)	0.250
Heart diseases	0.61 (0.36, 1.03)	0.064	0.69 (0.43, 1.11)	0.125
Asthma	1.00 (0.78, 1.29)	0.976	1.20 (0.95, 1.50)	0.121
Other lung diseases	1.14 (0.46, 2.81)	0.775	1.19 (0.54, 2.63)	0.667
Rheumatological diseases	0.77 (0.56, 1.05)	0.102	0.88 (0.67, 1.17)	0.377
Neurological diseases	1.55 (0.78, 3.10)	0.215	1.17 (0.65, 2.09)	0.601
Diabetes	1.02 (0.73, 1.42)	0.909	0.87 (0.66, 1.16)	0.337
Moderna vs. Pfizer-BioNTech	2.89 (2.39, 3.49)	**<0.001**	2.71 (2.32, 3.17)	**<0.001**
Prior COVID-19	0.67 (0.48, 0.95)	**0.024**	0.89 (0.65, 1.23)	0.474
Local reaction post Dose 1	9.09 (7.70, 10.73)	**<0.001**	1.08 (0.90, 1.28)	0.406
Systemic reaction post Dose 1	1.07 (0.89, 1.28)	0.475	2.46 (2.10, 2.89)	**<0.001**
Local reaction post Dose 2	-	-	1.74 (1.47, 2.05)	**<0.001**

The adjusted odd ratio (OR) and 95% confidence interval (CI) are presented. For categorical variables with more than two categories, the reference level is indicated. The analysis is by binominal logistic regression.

Regarding reaction severity following the second vaccine dose, younger age groups (<60 years), female sex, Asian race, Moderna brand, and first-dose systemic reactions were associated with more severe local reactions. Prior COVID-19 was associated with less severe local reactions. Younger age (<60 years), female sex, Asian race, history of food or drug allergy, having an epinephrine auto-injector, Moderna vaccine, and first-dose systemic reactions were associated with more severe systemic reactions following the second dose. Conversely, individuals with prior COVID-19 or reported second-dose local reactions experienced less severe systemic reactions following the second dose (**Table 7**). The severity of local and systemic reaction following the second dose according to the different participants characteristics are summarized in **S6 Table**.

**Table 7:** Factors associated with the severity of local and systemic reactions after the second dose of the COVID-19 vaccine.

	Local reaction severity		Systemic reaction severity	
Characteristic	Mdn dif. (95% CI)	*p* value	Mdn dif. (95% CI)	*p* value
Age (yrs.), 18–24 vs. 60 plus	0.6 (0.2, 1)	**0.003**	1 (0.46, 1.54)	**<0.001**
Age (yrs.), 25–39 vs. 60 plus	0.9 (0.63, 1.17)	**<0.001**	1 (0.62, 1.38)	**<0.001**
Age (yrs.), 40–59 vs. 60 plus	0.6 (0.37, 0.83)	**<0.001**	0.67 (0.29, 1.04)	**<0.001**
Sex, female vs. male	0.6 (0.41, 0.79)	**<0.001**	0.33 (0.1, 0.57)	**0.006**
Race, Black vs. White	0.6 (-0.05, 1.25)	0.071	1 (-0.34, 2.34)	0.144
Race, Asian vs. White	0.6 (0.24, 0.96)	**0.001**	1 (0.48, 1.52)	**<0.001**
Race, Other vs. White	0.4 (-0.14, 0.94)	0.147	0.67 (-0.08, 1.42)	0.081
Hispanic ethnicity	-0.1 (-0.66, 0.46)	0.727	0 (-0.65, 0.65)	1.000
Food allergy	0 (-0.39, 0.39)	1.000	0.67 (0.26, 1.07)	0**.001**
Drug allergy	-0.2 (-0.42, 0.02)	0.078	0.33 (0.04, 0.63)	**0.027**
Bee sting allergy	0.3 (-0.31, 0.91)	0.336	0 (-0.63, 0.63)	1.000
Allergy to other vaccines	-0.4 (-2.19, 1.39)	0.661	1 (-0.7, 2.7)	0.248
Epinephrine autoinjector	0 (-0.61, 0.61)	1.000	-0.67 (-1.48, 0.15)	0.108
Heart diseases	0 (-0.64, 0.64)	1.000	0.67 (-0.17, 1.5)	0.117
Asthma	0 (-0.31, 0.31)	1.000	0 (-0.38, 0.38)	1.000
Other lung diseases	0.6 (-0.87, 2.07)	0.425	0.67 (-0.77, 2.1)	0.362
Rheumatological diseases	0.2 (-0.25, 0.65)	0.379	0.67 (-0.23, 1.56)	0.145
Neurological diseases	0.4 (-0.13, 0.93)	0.136	0.33 (-0.84, 1.51)	0.578
Diabetes	0.2 (-0.13, 0.53)	0.228	-0.33 (-0.78, 0.11)	0.142
Moderna vs. Pfizer-BioNTech	1 (0.81, 1.19)	**<0.001**	1 (0.78, 1.22)	<0.001
Prior COVID-19	-0.7 (-1.11, -0.29)	**0.001**	-0.67 (-1.26, -0.07)	**0.028**
Local reaction post Dose 1	0.2 (-0.08, 0.48)	0.162	0 (-0.26, 0.26)	1.000
Systemic reaction post Dose 1	0.4 (0.21, 0.59)	**<0.001**	0.67 (0.4, 0.94)	**<0.001**
Local reaction post Dose 2	-	-	-0.33 (-0.63, -0.03)	**0.030**

The adjusted Mdn. dif. (Median difference) and 95% confidence interval (CI) are presented. For baseline characteristics with more than two categories, the reference category is indicated. The analysis is by quantile regression of the median.

## Discussion

In this survey of multi-site healthcare system employees, we explored factors associated with vaccine hesitancy and reactogenicity during the early phase of vaccine rollout. Females, Black individuals, and those who are allergic to other vaccines or working in a non-clinical setting were less likely to opt in for vaccination. Females and Asians were more likely to experience vaccine reactions, while older individuals experienced less reactogenicity. Moderna vaccine was associated with more reactogenicity when compared to the Pfizer-BioNTech vaccine. Prior COVID-19 predicted more severe reactions following the first but not the second dose. Lastly, local reactions predicted systemic reaction occurrence. These findings are of importance as we continue to struggle to increase the COVID-19 vaccination rate amid a growing vaccine hesitancy [13]. Identification of these factors can assist in designing targeted vaccine campaigns and empower providers counseling patients about COVID-19 vaccine expectations. Further, our findings may provide lessons for enhancing acceptance of future vaccines.

Our finding relating the Black race to vaccine hesitancy highlights another aspect of the COVID-19 pandemic disparity. Prior to vaccine availability, a survey of US adults about their intent to receive the COVID-19 vaccine similarly showed that the Black race was independently associated with vaccine hesitancy [9]. Interestingly, the same study showed that Asians were more likely to elect for vaccination, which agrees with our findings [9]. Another report by Stoler *et al*. showed that lack of medical trust is the primary reason driving vaccine hesitancy in the Black population [14]. Besides racial differences in vaccine hesitancy, participants with asthma, or allergy to other vaccines were less likely to receive the vaccine. Our study which focused on US patients diverges from the findings of a multinational survey study of low-to-middle income countries which showed that respiratory diseases were associated with a higher willingness to pay for the COVID-19 vaccine [15]. This discrepancy may be explained by the latter multinational study not distinguishing between asthma and other lung diseases, as other lung diseases were not associated with vaccine hesitancy in our study [15].

While further studies are needed to determine whether individuals with asthma or allergy to other vaccines are at increased risk of reactogenicity, our study showed that allergic co-morbidities are associated with higher odds of systemic reactions, particularly of allergic nature which is in agreement with prior observations of another survey-based study [16]. However, anaphylaxis was a rare reaction reported by only six participants in our study. In a CDC report on COVID-19 vaccine safety, there were 4.5 cases per million Pfizer-BioNTech and 2.5 cases per million Moderna vaccine doses administered, with most cases reported in females, following the first dose, and in those with food or drug allergy history [17, 18].

An interesting study that surveyed participants about their willingness to take four different hypothetical vaccines with various degrees of efficacy and safety, showed that these two factors do influence vaccine acceptance with individuals willing to trade higher efficacy for more safe vaccines [19]. In line with these observations, concerns about side effects and doubts about vaccine effectiveness were the most common reasons cited by study participants for electing not to receive the vaccine in our study.

Consistent with previous reports [17, 20], our study provides reassurance that older individuals, particularly those older than 60 years, are less likely to experience local or systemic reactions and incur less severe reactions. Another reassuring finding is that medical co-morbidities, unlike allergic comorbidities, do not influence vaccine reactogenicity. On the contrary, we showed that females are more likely to experience local and systemic reactions after the first or second vaccine dose and to endure more severe reactions. In line with this observation, the majority of the adverse events reported to the CDC were by females [18, 21]. Additionally, we found Asians to report more severe reactions, particularly systemic reactions, a finding that warrants further investigation.

In this study, the Moderna vaccine was associated with higher odds and greater severity of both vaccine reactions, particularly following the second vaccine dose. Such differences have been suggested by systematic review of clinical trials and real-world data [12, 20]. While the differences observed in the reactogenicity between the two vaccines were small, the side effects explored in this study were subjective, hence, their clinical significance is difficult to ascertain especially given that we did not provide guidance to participants on how to best respond to the severity scale.

A prior small-scale study showed that vaccine recipients with prior COVID-19 develop more systemic reactions than those without preexisting immunity; however, there were no differences in the occurrence of local reactions [22]. In this study, prior COVID-19 was associated with more severe and higher odds of both local and systemic reactions after the first vaccine dose. The effect was more prominent for systemic reactions following the first dose whereby participants with prior COVID-19 had ~4 times the odds of systemic reactions. This effect was isolated to the first vaccine dose as prior COVID-19 was associated with lower odds of reactogenicity after the second dose. This latter observation may explain the lower rate of systemic reaction among recipients of the third dose of the mRNA-based COVID-19 vaccine [23].

As vaccine efficacy is dependent upon successful receipt of two or more doses, and since some individuals experienced severe reactions after the first dose, information on whether such reactions to the first dose would inform the likelihood of local or systemic reactions with subsequent vaccine dose is of interest. Here, we showed that local reactions predicted the occurrence of systemic reactions after each dose. More importantly, individuals who developed systemic reactions after the first dose were more likely to develop severe systemic reactions after the second vaccine dose.

We acknowledge several limitations to our study: 1) the survey-based design carries the potential for both response and recall bias, 2) the survey collection tool did not offer detailed instructions on how to best respond to questions and therefore variation in response by participants might have influenced our results, 3) In our examination of factors associated with vaccine hesitancy, we used broad occupational categories which might have overlooked the confounding effect of a precise occupation on the interaction between demographics and vaccine hesitancy, and 4) the reliance on an electronic delivery method might have posed a barrier to participation. Nonetheless, we used a large cohort from multiple affiliated healthcare organizations, timed the survey to the vaccine rollout, and used multiple reminders to enhance the response rate.

This study examined factors associated with hesitancy towards receiving the COVID-19 vaccines during the early rollout phase. We believe these factors are potentially similar to those we might face with any new vaccine rollout in the future and thus need to be considered and acted upon early to ensure more acceptance of new vaccines. Overall, we showed that the occurrence and severity of reactions following the mRNA-based COVID-19 vaccine vary by age, gender, race, vaccine brand, and prior COVID-19 status. These factors should be considered when counseling patients and weighed against the proven efficacy of the vaccine. As the success of mRNA-based COVID-19 vaccines can pave the way for future deployment of other mRNA-based vaccines, factors informing reactogenicity may serve to guide future mRNA-based vaccine research [24, 25]. Lastly, and in hope of attaining herd immunity, targeted educational campaigns for populations at higher risk of vaccine hesitancy including Black individuals, young age groups, and those with allergic co-morbidities remain an unmet need.

## Supporting information

S1 Fig Correlation between local and systemic reaction severity. Heat map showing the Pearson correlation coefficients between local (LR) and systemic (SR) reaction severity following the first and second vaccine dose.
(TIF)

S1 Table Baseline characteristics of vaccine receivers versus non-receivers. Variables are expressed as n (%). Statistical significance was assessed using the chi-square test.
(DOCX)

S2 Table Reason(s) cited by participants for electing not to receive the first or second vaccine dose. Participants were able to pick more than one reason for electing not to receive the vaccine.
(DOCX)

S3 Table Baseline characteristics of individuals who received the Moderna or Pfizer-BioNTech vaccine. Baseline characteristics are expressed as n (%). Statistical significance was assessed using the chi-square test.
(DOCX)

S4 Table Characteristics of participants with reported anaphylaxis to COVID-19 vaccines. Abbreviations: LR, local reaction; SR, systemic reaction.
(DOCX)

S5 Table Severity of the local and systemic reactions after the first vaccine dose. The median (Mdn) and interquartile range (IQR) are presented. Statistical significance was assessed using Kruskal–Wallis for multiple groups comparisons and the Mann-Whitney *U* test for two groups comparisons.
(DOCX)

S6 Table Severity of local and systemic reactions after second vaccine dose. The median (Mdn) and interquartile range (IQR) are presented. Statistical significance was assessed using Kruskal–Wallis for multiple groups comparisons and the Mann-Whitney *U* test for two groups comparisons.
(DOCX)

S1 File Dataset. (XLSX)

S2 File Survey. (PDF)
