# Supplementary figures and images for: Hesitancy and reactogenicity to mRNA-based COVID-19 vaccines–Early experience with vaccine rollout in a multi-site healthcare system
Source: PLoS One. 2022 Aug 5;17(8):e0272691. doi: 10.1371/journal.pone.0272691 (PMC9355214; doi:10.1371/journal.pone.0272691)

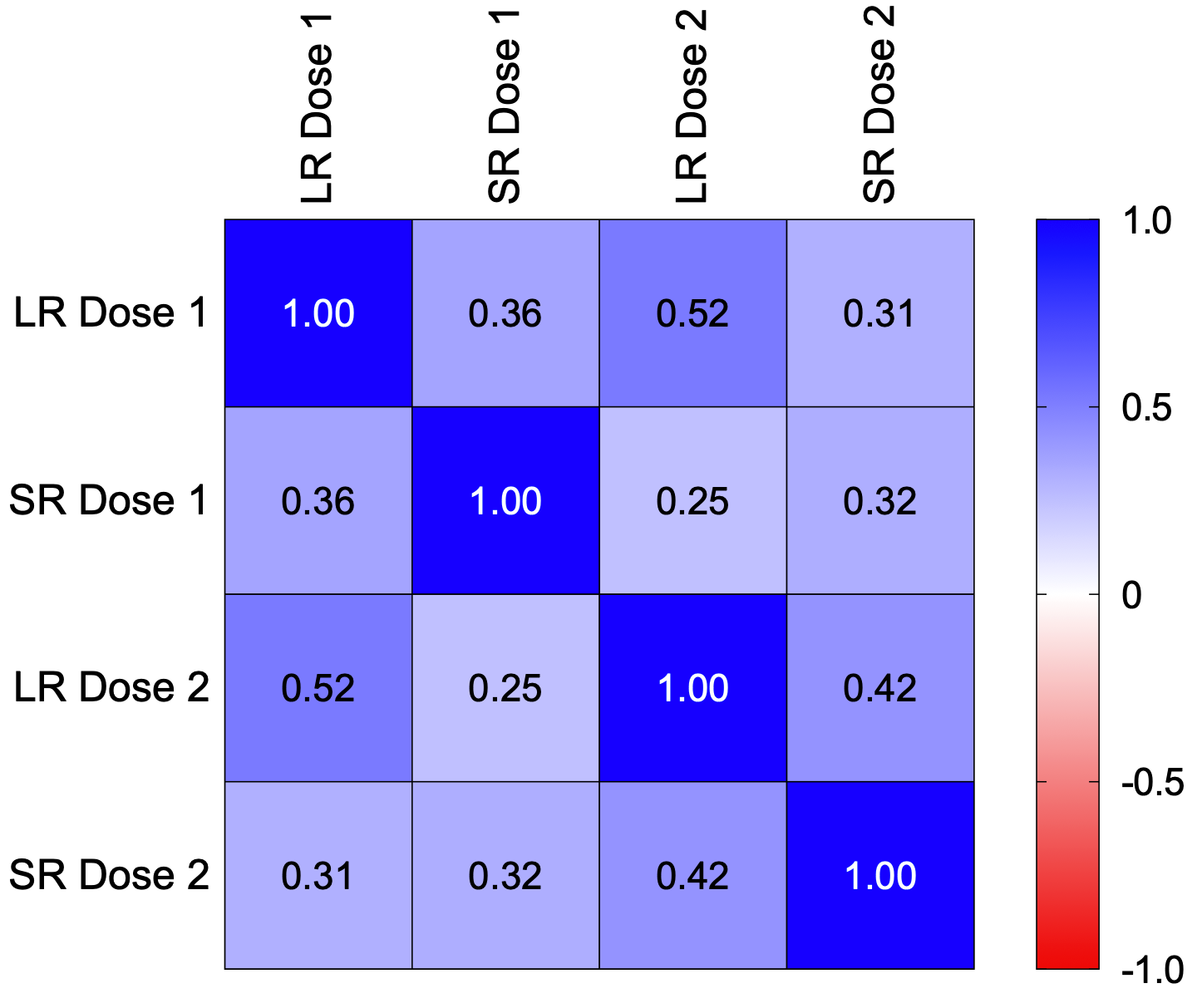

Supplement: S1 Fig — Heat map showing the Pearson correlation coefficients between local (LR) and systemic (SR) reaction severity following the first and second vaccine dose. (TIF) [file pone.0272691.s001.tif]
