# Supplementary material for: Hesitancy and reactogenicity to mRNA-based COVID-19 vaccines–Early experience with vaccine rollout in a multi-site healthcare system
Source: PLoS One. 2022 Aug 5;17(8):e0272691. doi: 10.1371/journal.pone.0272691 (PMC9355214; doi:10.1371/journal.pone.0272691)
Supplement: S1 Table — Variables are expressed as n (%). Statistical significance was assessed using the chi-square test. (DOCX) [file pone.0272691.s002.docx]

**S1 Table. Baseline characteristics of vaccine receivers versus non-receivers**

| **Characteristic** | **Vaccine receiver**  **(N=5340)** | **Non-vaccine receiver**  **(N=369)** | ***p* value** |
| --- | --- | --- | --- |
| **Age^a^** |  |  | **0.001** |
| 18-24 | 272 (5.15) | 19 (5.10) |  |
| 25-39 | 1863 (34.93) | 150 (40.65) |  |
| 40-59 | 2391(44.83) | 171 (46.34) |  |
| 60 plus | 807 (15.13) | 29 (7.86) |  |
| **Sex^a^** |  |  | **<0.001** |
| Male | 1180 (22.22) | 53 (14.56) |  |
| Female | 4130 (77.78) | 311(85.44) |  |
| **Race^a^** |  |  | **<0.001** |
| White | 4711 (90.09) | 312 (89.14) |  |
| Black | 84 (1.61) | 17 (4.86) |  |
| Asians | 295 (5.64) | 7(2.00) |  |
| Other | 139 (2.66) | 14 (4.00) |  |
| **Ethnicity^a^** |  |  | 0.102 |
| Hispanic | 157 (3.02) | 16 (4.60) |  |
| Non-Hispanics | 5037 (96.98) | 332 (95.40) |  |
| **Allergic co-morbidities** |  |  |  |
| Food allergy | 455 (8.52) | 57 (15.45) | **<.001** |
| Drug allergy | 1275 (23.88) | 98 (26.56) | 0.244 |
| Bee sting allergy | 230 (4.31) | 22 (5.96) | 0.134 |
| Allergy to other vaccine | 50 (0.94) | 30 (8.13) | **<.001** |
| Asthma | 608 (11.39) | 66 (17.89) | **<.001** |
| Epinephrine autoinjector | 195 (3.65) | 25 (6.78) | 0.003 |
| **Medical co-morbidities**  Heart diseases | 97 (1.82) | 8 (2.17) | 0.627 |
| Other lung diseases | 39 (0.73) | 3 (0.81) | 1.000 |
| Rheumatological disease | 323 (6.05) | 28 (7.59) | 0.234 |
| Neurological disease | 75 (1.40) | 9 (2.44) | 0.110 |
| Diabetes Mellitus | 295 (5.52) | 21 (5.69) | 0.892 |

Variables are expressed as n (%). Statistical significance was assessed using the chi-square test.

^a^Age information was missing from 7 participants; sex information was missing from 35 participants; race information was missing from 130 participants; ethnicity information was missing from 167 participants.
