# Supplementary material for: Hesitancy and reactogenicity to mRNA-based COVID-19 vaccines–Early experience with vaccine rollout in a multi-site healthcare system
Source: PLoS One. 2022 Aug 5;17(8):e0272691. doi: 10.1371/journal.pone.0272691 (PMC9355214; doi:10.1371/journal.pone.0272691)
Supplement: S2 Table — Participants were able to pick more than one reason for electing not to receive the vaccine. (DOCX) [file pone.0272691.s003.docx]

**S2 Table. Reason(s) cited by participants for electing not to receive the first or second vaccine dose**

| Reason(s) cited for electing not to receive the vaccine | N. | % |
| --- | --- | --- |
| I am worried about side effects - I need long term safety data | 212 | 57.5% |
| Other reasons^a^ | 118 | 32.0% |
| I don’t believe in the effectiveness of the COVID-19 vaccine | 75 | 20.3% |
| I was infected with COVID-19; therefore, I don’t think I need the vaccine | 48 | 13.0% |
| I’m pregnant or planning to become pregnant | 45 | 12.2% |
| I have allergy to other vaccines | 14 | 3.8% |
| I have allergy to vaccine component | 8 | 2.2% |
| I have immunodeficiency, so the vaccine will not work for me | 8 | 2.2% |
| I don’t have time to schedule a visit to receive the vaccine | 7 | 1.9% |
| I’m receiving immunosuppressive medication, so the vaccine will not work for me. | 6 | 1.6% |
| The vaccine was not offered to me | 5 | 1.4% |
| Reason(s) cited for electing not to receive the second vaccine dose | **N** | **%** |
| My dose is not due yet | 91 | 68.9% |
| The reaction after the first vaccine was so intense, I don’t want the second one at all | 9 | 6.8% |
| The reaction after the first vaccine was so intense, I will wait longer to have the second vaccine | 4 | 3.0% |
| I had COVID-19 so don’t think I need a second vaccine | 3 | 2.3% |
| It has been longer than 3 weeks since my last vaccination, but it hasn’t been offered to me | 2 | 1.5% |
| I haven’t had time to get the second vaccine | 3 | 2.3% |
| Other reasons | 33 | 25.0% |

Participants were able to pick more than one reason for electing not to receive the vaccine.

^a^Most common other reason cited was breastfeeding.
