# Supplementary material for: Hesitancy and reactogenicity to mRNA-based COVID-19 vaccines–Early experience with vaccine rollout in a multi-site healthcare system
Source: PLoS One. 2022 Aug 5;17(8):e0272691. doi: 10.1371/journal.pone.0272691 (PMC9355214; doi:10.1371/journal.pone.0272691)
Supplement: S3 Table — Baseline characteristics are expressed as n (%). Statistical significance was assessed using the chi-square test. (DOCX) [file pone.0272691.s004.docx]

**S3 Table. Baseline characteristics of individuals who received the Moderna or Pfizer-BioNTech vaccine**

| **Characteristic** | **Pfizer-BioNTech**  **(N=3469)** | **Moderna**  **(N=1871)** | ***p* value** |
| --- | --- | --- | --- |
| **Age (yrs)^a^** |  |  | **<0.001** |
| 18-24 | 131 (3.78) | 141 (7.55) |  |
| 25-39 | 1230 (35.49) | 633 (33.90) |  |
| 40-59 | 1563 (45.10) | 828 (44.35) |  |
| 60 plus | 542 (15.64) | 265 (14.19) |  |
| **Sex^a^** |  |  | **0.032** |
| Male | 735 (21.32) | 445 (23.89) |  |
| Female | 2712 (78.68) | 1418 (76.11) |  |
| **Race^a^** |  |  | **0.004** |
| White | 3088 (91.04) | 1623 (88.35) |  |
| Black | 54 (1.59) | 30 (1.63) |  |
| Asian | 163 (4.81) | 132 (7.19) |  |
| Other | 87 (2.56) | 52 (2.83) |  |
| **Ethnicity^a^** |  |  | 0.237 |
| Hispanic | 109 (3.23) | 48 (2.64) |  |
| Non-Hispanic | 3267 (96.77) | 1770 (97.36) |  |
| **Allergic co-morbidities** |  |  |  |
| Food allergy | 304 (8.76) | 151 (8.07) | 0.387 |
| Drug allergy | 856 (24.68) | 419 (22.39) | 0.062 |
| Bee sting allergy | 136 (3.92) | 94 (5.02) | 0.058 |
| Allergy to other vaccine | 32 (0.92) | 18 (0.96) | 0.886 |
| Asthma | 383 (11.04) | 225 (12.03) | 0.280 |
| Epinephrine autoinjector | 124 (3.57) | 71 (3.79) | 0.682 |
| **Medical co-morbidities** |  |  |  |
| Heart disease | 64 (1.84) | 33 (1.76) | 0.832 |
| Other Lung diseases (e.g., COPD) | 23 (0.66) | 16 (0.86) | 0.431 |
| Rheumatological disease | 206 (5.94) | 117 (6.25) | 0.645 |
| Neurological disease | 46 (1.33) | 29 (1.55) | 0.507 |
| Diabetes mellitus | 187 (5.39) | 108 (5.77) | 0.560 |

Baseline characteristics are expressed as n (%). Statistical significance was assessed using the chi-square test.

^a^Age information was missing from 7 participants; sex information was missing from 30 participants; race information was missing from 111 participants; ethnicity information was missing from 146 participants.
