# Supplementary material for: Hesitancy and reactogenicity to mRNA-based COVID-19 vaccines–Early experience with vaccine rollout in a multi-site healthcare system
Source: PLoS One. 2022 Aug 5;17(8):e0272691. doi: 10.1371/journal.pone.0272691 (PMC9355214; doi:10.1371/journal.pone.0272691)
Supplement: S4 Table — Abbreviations: LR, local reaction; SR, systemic reaction. (DOCX) [file pone.0272691.s005.docx]

**S4 Table. Characteristics of participants with reported anaphylaxis to COVID-19 vaccines**

|  | **Participant #1*** | **Participant #2** | **Participant #3** | **Participant #1^a^** | **Participant #4** | **Participant #5** |
| --- | --- | --- | --- | --- | --- | --- |
| **Age** | 25-39 | 60-79 | 40-59 | 25-39 | 40-59 | 60-79 |
| **Sex** | Female | Female | Female | Female | Male | Female |
| **Race** | White | White | White | White | White | White |
| **Other allergies** | None | food & drug allergy, Asthma | Food, drug allergy & bee sting allergy, asthma | None | None | food, drug & other vaccines allergies, asthma |
| **Medical co-morbidities** | None | None | Rheumato-logical diseases | None | None | Rheumato-logical diseases |
| **Prior COVID-19** | No | No | No | No | No | No |
| **Vaccine brand** | Moderna | Pfizer-BioNTech | Moderna | Moderna | Moderna | Moderna |
| **LR post dose 1** | Yes | No | No | Yes | No | Yes |
| **SR post dose 1** | Yes | Yes | Yes | Yes | No | Yes |
| **Received dose 2** | Yes | No | No | Yes | Yes | Yes |
| **LR post dose 2** | Yes | - | - | Yes | No | Yes |
| **SR post dose 2** | Yes | - | - | Yes | Yes | Yes |

Abbreviations: LR, local reaction; SR, systemic reaction

^a^Participant #1 indicated having anaphylaxis after the first and second vaccine dose.
