# Supplementary material for: Hesitancy and reactogenicity to mRNA-based COVID-19 vaccines–Early experience with vaccine rollout in a multi-site healthcare system
Source: PLoS One. 2022 Aug 5;17(8):e0272691. doi: 10.1371/journal.pone.0272691 (PMC9355214; doi:10.1371/journal.pone.0272691)
Supplement: S5 Table — The median (Mdn) and interquartile range (IQR) are presented. Statistical significance was assessed using Kruskal–Wallis for multiple groups comparisons and the Mann-Whitney U test for two groups comparisons. (DOCX) [file pone.0272691.s006.docx]

**S5 Table. The severity of the local and systemic reactions after the first vaccine dose**

|  | **Local reactions** | |  | **Systemic reactions** | |
| --- | --- | --- | --- | --- | --- |
| **Characteristic** | **Mdn (IQR)** | ***p* value** |  | **Mdn (IQR)** | ***p* value** |
| **Age (yrs.)** |  | **<0.001** |  |  | 0.163 |
| 18-24 | 5 (3-7) |  |  | 5 (3-6) |  |
| 25-39 | 4 (3-6) |  |  | 5 (3-7) |  |
| 40-59 | 4 (3-6) |  |  | 5 (3-7) |  |
| 60 plus | 3 (2-5) |  |  | 4 (3-6) |  |
| **Gender** |  | **<0.001** |  |  | 0.191 |
| Male | 4 (2-5) |  |  | 4 (3-6) |  |
| Female | 4 (3-6) |  |  | 4 (3-7) |  |
| **Race** |  | **<0.001** |  |  | 0.263 |
| White | 4 (3-6) |  |  | 5 (3-6) |  |
| Black | 5 (3-6) |  |  | 5 (4-7) |  |
| Asian | 5 (3-6) |  |  | 5 (3-8) |  |
| Other/unknown | 4 (4-7) |  |  | 5 (3-7) |  |
| **Ethnicity** |  | **0.019** |  |  | 0.467 |
| Hispanic | 4 (3-7) |  |  | 5 (3-7) |  |
| Non-Hispanic | 4 (3-6) |  |  | 5 (3-6) |  |
| **Allergic co-morbidities** |  |  |  |  |  |
| Food allergy |  | 0.101 |  |  | 0.595 |
| Yes | 4 (3-6) |  |  | 5 (3-6) |  |
| No | 4 (3-6) |  |  | 5 (3-6) |  |
| Drug allergy |  | 0.873 |  |  | 0.108 |
| Yes | 4 (3-6) |  |  | 5 (3-6) |  |
| No | 4 (3-6) |  |  | 5 (3-6) |  |
| Bee sting allergy |  | 0.983 |  |  | 0.516 |
| Yes | 4 (3-6) |  |  | 5 (3-6) |  |
| No | 4 (3-6) |  |  | 5 (3-6) |  |
| Allergy to other vaccines |  | 0.700 |  |  | 0.649 |
| Yes | 4 (3-6) |  |  | 4 (3-6) |  |
| No | 4 (3-6) |  |  | 5 (3-6) |  |
| Asthma |  | 0.323 |  |  | 0.317 |
| Yes | 4 (3-6) |  |  | 5 (3-7) |  |
| No | 4 (3-6) |  |  | 5 (3-6) |  |
| Epinephrine autoinjector |  | 0.906 |  |  | 0.320 |
| Yes | 4 (3-6) |  |  | 5 (3-6) |  |
| No | 4 (3-6) |  |  | 5 (3-6) |  |
| **Medical co-morbidities** |  |  |  |  |  |
| Heart diseases |  | 0.836 |  |  | **0.056** |
| Yes | 4 (3-5) |  |  | 5 (4-7) |  |
| No | 4 (3-6) |  |  | 5 (3-6) |  |
| Other lung diseases |  | 0.927 |  |  | 0.300 |
| Yes | 3.5 (3-6) |  |  | 5 (4-7) |  |
| No | 4 (3-6) |  |  | 5 (3-6) |  |
| Rheumatological diseases |  | 0.298 |  |  | 0.074 |
| Yes | 4 (2-6) |  |  | 5 (3-8) |  |
| No | 4 (3-6) |  |  | 5 (3-6) |  |
| Neurological diseases |  | 0.956 |  |  | **0.015** |
| Yes | 4 (2-6) |  |  | 6 (4-8) |  |
| No | 4 (3-6) |  |  | 5 (3-6) |  |
| Diabetes mellitus |  | 0.962 |  |  | 0.511 |
| Yes | 4 (3-6) |  |  | 5 (3-7) |  |
| No | 4 (3-6) |  |  | 5 (3-6) |  |
| **Other Factors** |  |  |  |  |  |
| Vaccine brand |  | **<0.001** |  |  | 0.245 |
| Pfizer-BioNTech | 4 (2-5) |  |  | 4 (3-6) |  |
| Moderna | 4 (3-6) |  |  | 5 (3-7) |  |
| Prior COVID-19 |  | **<0.001** |  |  | **<0.001** |
| Yes | 5 (3-7) |  |  | 5 (4-7) |  |
| No | 4 (3-6) |  |  | 4 (3-6) |  |
| Local reaction post Dose 1 |  |  |  |  | 0.066 |
| Yes | - |  |  | 5 (3-7) |  |
| No |  |  |  | 4 (3-6) |  |

The median (Mdn) and interquartile range (IQR) are presented. Statistical significance was assessed using Kruskal–Wallis for multiple groups comparisons and the Mann-Whitney *U* test for two groups comparisons.
