# Supplementary material for: Hesitancy and reactogenicity to mRNA-based COVID-19 vaccines–Early experience with vaccine rollout in a multi-site healthcare system
Source: PLoS One. 2022 Aug 5;17(8):e0272691. doi: 10.1371/journal.pone.0272691 (PMC9355214; doi:10.1371/journal.pone.0272691)
Supplement: S6 Table — The median (Mdn) and interquartile range (IQR) are presented. Statistical significance was assessed using Kruskal–Wallis for multiple groups comparisons and the Mann-Whitney U test for two groups comparisons. (DOCX) [file pone.0272691.s007.docx]

**S6 Table. The severity of local and systemic reactions after second vaccine dose**

| **Characteristic** | **Local reactions** | |  | **Systemic reactions** | |
| --- | --- | --- | --- | --- | --- |
|  | **Mdn (IQR)** | ***p* value** |  | **Mdn (IQR)** | ***p* value** |
| **Age (yrs.)** |  | **<0.001** |  |  | **<0.001** |
| 18-24 | 4 (3-6) |  |  | 6 (5-8) |  |
| 25-39 | 4 (3-6) |  |  | 6 (4-8) |  |
| 40-59 | 4 (3-6) |  |  | 6 (4-8) |  |
| 60 plus | 3 (2-5) |  |  | 5 (4-7) |  |
| **Gender** |  | **<0.001** |  |  | **<0.001** |
| Male | 4 (2-5) |  |  | 5 (4-7) |  |
| Female | 4 (3-6) |  |  | 6 (4-8) |  |
| **Race** |  | **<0.001** |  |  | **<0.001** |
| White | 4 (2-6) |  |  | 6 (4-8) |  |
| Black | 4 (3-7) |  |  | 7 (4-8) |  |
| Asian | 4 (3-7) |  |  | 7 (4-8) |  |
| Other/unknown | 4 (3-7) |  |  | 7 (5-8.5) |  |
| **Ethnicity** |  | **0.016** |  |  | **0.038** |
| Hispanic | 4 (3-7) |  |  | 6.5 (4-8) |  |
| Non-Hispanic | 4 (3-6) |  |  | 6 (4-8) |  |
| **Allergic co-morbidities** |  |  |  |  |  |
| Food allergy |  | 0.401 |  |  | **<0.001** |
| Yes | 4 (3-6) |  |  | 7 (4-8) |  |
| No | 4 (3-6) |  |  | 6 (4-8) |  |
| Drug allergy |  | 0.861 |  |  | **0.012** |
| Yes | 4 (3-6) |  |  | 6 (4-8) |  |
| No | 4 (3-6) |  |  | 6 (4-8) |  |
| Bee sting allergy |  | **0.022** |  |  | 0.600 |
| Yes | 4 (3-7) |  |  | 6 (4-8) |  |
| No | 4 (3-6) |  |  | 6 (4-8) |  |
| Allergy to other vaccines |  | 0.780 |  |  | **0.037** |
| Yes | 4 (2-6) |  |  | 7 (4-9) |  |
| No | 4 (3-6) |  |  | 6 (4-8) |  |
| Asthma |  | 0.231 |  |  | **0.022** |
| Yes | 4 (3-6) |  |  | 6 (4-8) |  |
| No | 4 (3-6) |  |  | 6 (4-8) |  |
| Epinephrine autoinjector |  | 0.302 |  |  | 0.876 |
| Yes | 4 (3-6) |  |  | 6 (4-8) |  |
| No | 4 (3-6) |  |  | 6 (4-8) |  |
| **Medical co-morbidities** |  |  |  |  |  |
| Heart diseases |  | 0.549 |  |  | **0.032** |
| Yes | 4 (2-6) |  |  | 7 (5-8) |  |
| No | 4 (3-6) |  |  | 6 (4-8) |  |
| Other lung diseases |  | 0.694 |  |  | 0.445 |
| Yes | 4 (2-6) |  |  | 7 (4-8) |  |
| No | 4 (3-6) |  |  | 6 (4-8) |  |
| Rheumatological diseases |  | 0.458 |  |  | **0.014** |
| Yes | 4 (3-6) |  |  | 7 (4-8) |  |
| No | 4 (3-6) |  |  | 6 (4-8) |  |
| Neurological diseases |  | 0.305 |  |  | 0.166 |
| Yes | 4 (3-6) |  |  | 7 (4-8) |  |
| No | 4 (3-6) |  |  | 6 (4-8) |  |
| Diabetes mellitus |  | 0.865 |  |  | 0.294 |
| Yes | 4 (3-6) |  |  | 6 (4-8) |  |
| No | 4 (3-6) |  |  | 6 (4-8) |  |
| **Other Factors** |  |  |  |  |  |
| Vaccine brand |  | **<0.001** |  |  | **<0.001** |
| Pfizer-BioNTech | 4 (2-5) |  |  | 5 (4-8) |  |
| Moderna | 5 (3-7) |  |  | 6 (4-8) |  |
| Prior COVID-19 |  | 0.086 |  |  | 0.872 |
| Yes | 4 (2-5) |  |  | 6 (4-8) |  |
| No | 4 (3-6) |  |  | 6 (4-8) |  |
| Local reaction post Dose 1 |  | **0.007** |  |  | **0.002** |
| Yes | 4 (3-6) |  |  | 6 (4-8) |  |
| No | 4 (2-5) |  |  | 5.5 (4-7) |  |
| Systemic reaction post Dose 1 |  | **<0.001** |  |  | **<0.001** |
| Yes | 4 (3-6) |  |  | 6 (4-8) |  |
| No | 4 (2-6) |  |  | 6 (4-8) |  |
| Local reaction post Dose 2 |  |  |  |  | **0.014** |
| Yes | - |  |  | 6 (4-8) |  |
| No | - |  |  | 6 (4-8) |  |

The Median (Mdn) and interquartile range (IQR) are presented. Statistical significance was assessed using Kruskal–Wallis for multiple groups comparisons and the Mann-Whitney *U* test for two groups comparisons.
