# Supplementary material for: Hesitancy and reactogenicity to mRNA-based COVID-19 vaccines–Early experience with vaccine rollout in a multi-site healthcare system
Source: PLoS One. 2022 Aug 5;17(8):e0272691. doi: 10.1371/journal.pone.0272691 (PMC9355214; doi:10.1371/journal.pone.0272691)
Supplement: S2 File — (PDF) [file pone.0272691.s009.pdf]

Survey Questions

Age in years
- 18-24
- 25-39
- 40-59
- 60-79
- 80 plus

Sex
- Male
- Female
- Prefer not to answer

What is your race?
- Caucasian
- African American
- Asian
- American Indian or Alaska Native
- Native Hawaiian or Other Pacific Islander
- Others
- Prefer not to answer

What is your ethnicity?
- Not Hispanic or Latino
- Hispanic or Latino
- Prefer not to answer

What is your job description?
- Physician
- Resident/Fellow Physician
- Advanced Practice Providers
- Nurse
- Student
- Researcher
- Technician
- Administration
- Care coordination/Social services
- IT staff
- Security
- Support staff
- Maintenance
- Other (Please specify)

Do you have any of the following? (Please select all that apply)
- Food allergy
- Drug allergy
- Bee sting allergy
- I had an allergic to other vaccines in the past I have an EpiPen prescription
- None of the above

Do you have an underlying medical condition?
- Heart disease (e.g. heart attack, heart failure)
- Asthma
  Other lung diseases (e.g. COPD)
- Rheumatologic (joint) disease (e.g., arthritis)
- Neurologic disease (e.g., stroke, multiple sclerosis)
- Diabetes
  None of the above.

Have you received the first dose of COVID-19 vaccine?

- Yes
- No

Which vaccine did you receive? (please check your vaccine card for this information)

- Pfizer-BioNtech
- Moderna
- Unable to find the information on the vaccination card

Have you been diagnosed with COVID-19 (you had a positive test for COVID-19) before you received the COVID-19 vaccine?

- Yes
- No

Did you develop a local injection site reaction (e.g. pain, muscle soreness, swelling, redness) after the first dose?

- Yes
- No

How would you rate the severity of your local reaction?

1 2 3 4 5 6 7 8 9 10

Did you develop other symptoms (OTHER than injection site reaction) after your first dose?

- Yes
- No

Which of the following other symptoms (OTHER than injection site reaction) have you developed? (please select all that apply)

- fever
- chills
- headache
- fatigue
- nausea
- vomiting
- diarrhea
- diffuse muscle pain
- diffuse joint pain
- Lymph node swelling tingling/numbness in your extremities extremity weakness tingling/numbness in your face
- facial palsy (facial weakness)
- facial swelling
- skin rash
- anaphylactic reaction requiring medical treatment other (Please specify)

Rate the severity of these other symptoms (OTHER than injection site reaction) that you experienced

1 2 3 4 5 6 7 8 9 10

Have you already received your second dose?

- Yes
- No

Did you develop a local injection site reaction (e.g., pain, muscle soreness, swelling, redness) after the second dose?

- Yes
- No

How would you rate the severity of your local reaction?

1 2 3 4 5 6 7 8 9 10

Did you develop other symptom(s) (OTHER than local or injection site reaction) after your second dose?

- Yes
- No

Which of the following other symptoms did you develop? (please select all that apply)

- fever
- chills
- headache
- fatigue
- nausea
- vomiting
- diarrhea
- diffuse muscle pain
- diffuse joint pain
- Lymph note swelling tingling/numbness in your extremities tingling/numbness in your face extremity weakness
- facial weakness
- facial swelling
- skin rash
- anaphylactic reaction requiring medical treatment other (Please specify)

Rate the severity of these other symptom(s) (OTHER than injection site reaction) you have experienced?

1 2 3 4 5 6 7 8 9 10

If you received your first vaccine, why haven't you received your second COVID-19 booster vaccine?

- My second dose is not due yet.
- The reaction after the first vaccine was so intense, I don't want the second one at all
- The reaction after the first vaccine was so intense, I will wait longer to have the second vaccine
- I had COVID-19 infection so don't think I need a second vaccine
- It has been longer than 3 weeks since my last vaccination, but it hasn't been offered to me.
- I haven't had time to get the second vaccine Other (Please specify in the box below)

Why haven't you received your first dose of COVID-19 vaccine? Please check all that apply

- I don't have time to schedule a visit to receive the vaccine
- I was infected with COVID-19; therefore, I don't think I need the vaccine.
- I don't believe in the effectiveness of the
- COVID-19 vaccine
- I'm worried about side effects - I need long term safety data
- I have allergy to vaccine component
- I have allergy to other vaccines
- I have immunodeficiency, so the vaccine will not work for me
- I'm receiving immunosuppressive medication, so the vaccine will not work for me.
- I'm pregnant or planning to become pregnant.
- The vaccine was not offered to me
- Other: please explain
